# Amlodipine-Associated Angioedema: An Integrated Pharmacovigilance Assessment Using Disproportionality and Interaction Analysis and Case Reviews

**DOI:** 10.3390/jcm14041097

**Published:** 2025-02-08

**Authors:** Kannan Sridharan, Gowri Sivaramakrishnan

**Affiliations:** 1 Department of Pharmacology & Therapeutics, College of Medicine & Health Sciences, Arabian Gulf University, Manama P.O. Box 26671, Bahrain; 2 Bahrain Defence Force Royal Medical Services, Riffa P.O. Box 28743, Bahrain; gowri.sivaramakrishnan@gmail.com

**Keywords:** amlodipine, ACEI, ARB, angioedema, aliskiren, pharmacovigilance

## Abstract

**Background:** Amlodipine has recently been incidentally reported with angioedema and is frequently prescribed with renin–angiotensin–aldosterone system inhibitors (RAAS-i) for hypertension management. While RAAS-i drugs are known to cause angioedema, the risk associated with amlodipine alone or in combination with RAAS-i drugs remains unclear. This study aimed to evaluate the association between amlodipine use and angioedema using pharmacovigilance data. **Methods:** We analyzed adverse event reports from the US FDA Adverse Event Reporting System using both frequentist and Bayesian approaches. Drug–drug interactions were assessed using multiplicative models. Additionally, we conducted a systematic review of published case reports of amlodipine-associated angioedema. **Results:** Among 29,661,136 reports, 2076 cases of angioedema were identified (1067 with amlodipine alone, 1009 with amlodipine–RAAS-i combinations). Significant safety signals were detected for amlodipine alone and in combination with aliskiren, specific ACE inhibitors (quinapril, benazepril, trandolapril, fosinopril, perindopril), and certain ARBs (candesartan, losartan). No significant interactions were observed between amlodipine and RAAS-i drugs except for the amlodipine–trandolapril combination. A review of published cases demonstrated definite causality in two cases and possible association in others, with most patients presenting with oropharyngeal/facial edema and achieving complete recovery following drug discontinuation and standard therapy. **Conclusions:** Our findings suggest a potentially increased risk of angioedema with amlodipine, both as monotherapy and in specific RAAS-i combinations. While these results should not discourage appropriate clinical use, they emphasize the importance of monitoring for angioedema, particularly during therapy initiation. The findings from this study need to be validated in prospective studies for further elucidation of the underlying mechanisms.

## 1. Introduction

Angioedema manifests as severe, painful swelling predominantly affecting the face, oropharyngeal tissues, and respiratory tract. This potentially life-threatening condition occurs through two primary mechanisms: mast cell activation or bradykinin release [1]. While various etiologies exist, medications represent a significant iatrogenic cause of angioedema [2]. Drug-associated angioedema was observed not only at the beginning of treatment but also while the patient was receiving drug therapy [3]. Angioedema episodes associated with drugs can be either allergic or non-allergic [4]. Allergic drug-induced angioedema is primarily induced by type 1 hypersensitivity reactions mediated by histamine, while non-allergic angioedema episodes are mediated by bradykinin [4]. A comprehensive analysis of the United States Food and Drug Administration Adverse Event Reporting System (USFDA AERS) database demonstrated the strongest positive signals for antithrombotic agents [reporting odds ratio (ROR) (95% CI): 22.53 (21.16–23.99)], followed by cardiovascular drugs [ROR (95% CI): 9.17 (8.87–9.48)] [2]. Among cardiovascular medications, angiotensin-converting enzyme inhibitors (ACEIs), angiotensin receptor blockers (ARBs), direct renin inhibitors (DRIs), neprilysin inhibitors, and tissue plasminogen activators are frequently implicated [5].

Calcium channel blockers represent one of the most widely prescribed classes of cardiovascular medications. They primarily block the long-acting voltage-gated calcium channels on the smooth muscles leading to vasodilation and are classified into dihydropyridines (DHPs) (acting predominantly on the blood vessels) and non-DHPs (cardiac conducting and contractile tissues). Among the DHPs, amlodipine stands out as a first-line antihypertensive agent, distinguished by its multiple pleotropic effects, including anti-atherosclerotic properties and renoprotection [6]. A key pharmacokinetic advantage of amlodipine is its low renal clearance, which confers a prolonged duration of action and sustained antihypertensive effects even with occasionally missed doses [7]. While generally well-tolerated, amlodipine’s established adverse effects include headache, lightheadedness, flushing, and peripheral edema, with the latter occurring in approximately 20–30% of patients [8]. A recent five-year retrospective analysis identified angioedema in 18 (9%) cases among calcium channel blocker recipients, with amlodipine accounting for 14 of these cases [9]. However, due to its rare occurrence, the magnitude of association between amlodipine and angioedema remains poorly characterized in the current literature.

The combination of amlodipine with renin–angiotensin–aldosterone system inhibitors (RAAS-i), particularly ACEIs, has demonstrated superior therapeutic outcomes compared to amlodipine monotherapy, including enhanced blood pressure control, reduced adverse events, and improved tolerability [10]. Recent clinical evidence further supports this synergistic approach, showing that amlodipine–ACEI combinations provide superior cardioprotection. This is evidenced by reduced incidence of cardiovascular mortality, myocardial infarction, stroke, angina-related hospitalizations, post-sudden cardiac death resuscitations, and coronary revascularization procedures, regardless of prior antihypertensive therapy [11]. Further, fixed dose combinations of amlodipine and valsartan (ARB) have been approved based on the evidence of superior antihypertensive efficacy compared to monotherapies [12]. Concomitant use of drugs poises instances for their effects to be modified, which can primarily be attributed to pharmacokinetic/pharmacodynamic alterations. The proportion of adverse events that can primarily be attributed to drug–drug interactions is around 30% [13]. In the pre-marketing period of the lifecycle of drugs, due to stringent eligibility criteria, patients receiving or required to receive multiple drugs are usually excluded. Further, the pre-marketing studies are also limited to excluding patients with multiple diseases as well as being associated with shorter durations. However, post-marketing, due to necessity, physicians may start using a combination of drugs for which there is a paucity of information available from the prior studies, due to which studies focusing on drug interactions are necessary for understanding the extent and impact of such drug interactions. While ACEIs are well-documented as the leading drug class associated with angioedema [2], with a recent study signaling a similar risk with ARBs [14], the potential risk of angioedema with concurrent ACEI/ARB and amlodipine therapy remains largely unexplored.

The USFDA AERS serves as a comprehensive repository of adverse event reports voluntarily submitted by healthcare professionals, consumers, and pharmaceutical manufacturers [15]. Pharmacovigilance through disproportionality analysis of these reports generates safety signals that warrant further investigation in real-world studies [16]. Given the limited understanding of both amlodipine-associated angioedema and potential interactions with RAAS-i drugs, we conducted a systematic analysis of USFDA AERS reports. Furthermore, a systematic review of published case reports relating amlodipine to angioedema was carried out to supplement the findings observed from the disproportionality analysis of the USFDA AERS. Our study aims to characterize the association between amlodipine and angioedema, while specifically examining the impact of concurrent RAAS-i therapy on this adverse event.

## 2. Methods

### 2.1. Data Source

Data pertaining to this study were obtained from the USFDA AERS, using the Standardised MedDRA (Medical Dictionary for Regulatory Activities) Query (Narrow) Term “Angioedema” (MedDRA code: 20000024) [17]. The MedDRA was developed by the International Council for Harmonisation of Technical Requirements for Pharmaceuticals for Human Use that encompasses specific and standardized medical terminologies that were to be implemented and reported by drug manufacturers, healthcare personnel, and consumers for sharing regulatory information related to pharmaceuticals, biologics, vaccines, and drug–device combination products. We obtained data relating to the adverse event reports submitted to USFDA AERS that were submitted between the first quarter of 2004 until the third quarter of 2024, encompassing a total of 83 quarterly reports.

### 2.2. Data Processing

The USFDA AERS was systematically searched for reports involving amlodipine as well as its combinations with RAAS-i drugs to ensure comprehensive retrieval of Individual Case Safety Reports (ICSRs) [18]. The search strategy details can be found in the Electronic Supplementary Table S2. The following RAAS-i drugs were evaluated in this study: direct renin inhibitor (DRI) (aliskiren), ACEIs (enalapril, benazepril, enalaprilat, captopril, moexipril, perindopril, trandolapril, fosinopril, quinapril, lisinopril, and ramipril), and ARBs (irbesartan, olmesartan, azilsartan, irbesartan, candesartan, losartan, eprosartan, telmisartan and valsartan). For assessing amlodipine (without RAAS-i drugs)-associated angioedema, we excluded reports containing DRI, ACEIs, or ARBs. As a part of the deduplication process, we adhered to the US FDA’s deduplication guidelines, where we sorted out the adverse event reports in ascending order using their Case_IDs, and we retained only those reports with the latest ICSR number, representing the most recent entry. The adverse event reports associated with angioedema with the drug/s of interest were first sorted out based on their type of association with the suspected adverse event. Reports were included in the final analysis only if they identified amlodipine as the “primary suspect” drug in association with angioedema. Reports associating angioedema to amlodipine use as secondary suspect or interacting or concomitant roles were excluded. Following this screening, the reports were arranged numerically in an order based on their Case_IDs, and reports containing duplicated Case_IDs were removed. Following this, reports containing duplicated FDA_DT reports were excluded and only those with unique ICSR numbers. In the search strategy used in this study, the search was limited to the non-proprietary drug names for amlodipine and its combinations. We obtained the following details from the adverse event reports: age, gender, report year, and reporting country.

### 2.3. Data Mining Algorithms

A “case–non-case” disproportionality analysis method was employed to evaluate the association of amlodipine (and its combinations) with angioedema by comparing the frequency of reports related to angioedema involving amlodipine compared to the reported frequency with all other drugs [19]. This statistical approach compares the frequencies of adverse events observed versus expected with the drug/s of interest compared to adverse events reported with all other drugs in the database aiding the detection of safety signals which can be explored in further studies. This statistical approach is the core of signal detection analysis in the pharmacovigilance database, serving as a vital tool in post-market drug safety surveillance. We retrieved and analyzed the data using the OpenVigil 2.1 platform for amlodipine–angioedema pairs. Two algorithms each were obtained pertaining to frequentist and Bayesian approaches for detecting potential safety signals related to amlodipine-associated angioedema.

The Reporting Odds Ratio (ROR) and the Proportional Reporting Ratio (PRR) were the signal detection measures in the frequentist category. The ROR was estimated as the ratio of odds of reporting angioedema with amlodipine/RAAS-i drugs or their combinations to all other adverse events over the same odds with all other drugs. Similarly, the PRR was estimated as the ratio of the proportion of adverse event reports with angioedema to all other adverse events with amlodipine/RAAS-i drugs or their combinations over all other drugs for the same proportion. The RORs in pharmacovigilance studies are the equivalent of odds ratios in the observational studies, such as case–control and cohort studies. Similarly, the PRR is equivalent to the relative risk used in prospective studies. These measures are based on the fundamentals of expected and observed numbers of reports with specific adverse events in the pharmacovigilance database. Evan’s signal detection criteria were adhered, where a minimum of three reports, a PRR > 2, and a chi-square (χ^2^) statistic > 4 for the drug–angioedema pair was considered as a signal [20]. A 95% confidence interval (CI) was calculated for both the ROR and PRR. The χ^2^ value corresponds to a *p*-value of <0.05, indicating that there is more than 95% probability that the observed numbers of adverse event reports with the drug/s of interest are different from the expected in the real-world scenario. The Relative Reporting Ratio (RRR) was estimated as the ratio between the observed cases of angioedema over the expected cases with amlodipine.

The Bayesian Confidence Propagation Neural Network (BCPNN) and the Multi-Item Gamma Poisson Shrinker (MGPS) formed the Bayesian analyses. The Information Component (IC), the logarithmic ratio of the observed co-occurrence of amlodipine and angioedema relative to the expected frequencies in the database, formed the signal detection measure in the BCPNN, and when the lower boundary of the 95% CI (IC025) exceeded zero, it was considered as a signal. The Empirical Bayes Geometric Mean (EBGM) was the signal detection measure in the MGPS, and a signal was considered to be detected if the lower boundary of the EBGM’s 95% CI limit (EBGM05) exceeded 2 [21,22]. Both frequentist and Bayesian measures of signal detection are sensitive in picking up adverse event signals, and the predictive accuracy can be improved by using a combination approach.

### 2.4. Interaction Signal Scores

The interaction strength between amlodipine and RAAS-i drugs for the risk of angioedema was evaluated using a multiplicative drug–drug interaction model [23]. The interaction model analyzes the extent of reporting the adverse event of interest with the drug combinations relative to expected numbers and is compared to the similar measure for each of the drugs individually. In the multiplicative drug–drug interaction model, the null hypothesis is assumed to be as follows: the proportion of patients encountering angioedema episodes with amlodipine and RAAS-i drugs when administered concomitantly is like the proportion receiving either of the drugs individually. The interaction formula that was used for assessing the potential interaction between amlodipine and RAAS-i drugs is outlined in Table 1. Both log-linear and logistic regression analyses were employed and e^β12^ (log risk of angioedema with amlodipine–RAAS-i drug combinations) and e^γ12^ (logit risk of angioedema with amlodipine–RAAS-i drug combinations) were estimated. When e^β12^ or e^γ12^ exceeds 1, an interaction signal was generated [23].

### 2.5. Outcomes Assessed

For the amlodipine and amlodipine–RAAS-i drug combination–rhabdomyolysis pairs, death, life-threatening events, and hospitalization were considered as the key outcomes.

### 2.6. Compliance with Reporting Standards

We adhered to the reporting standards laid down in the Reporting of a Disproportionality Analysis for drUg Safety signal detection using spontaneously reported adverse events in Pharmacovigilance (READUS-PV) [24].

### 2.7. Case Review

A comprehensive literature review was carried out in PubMed, Cochrane CENTRAL, and Google Scholar to identify case reports relating angioedema with amlodipine use. The search terms used were “amlodipine” [tiab] AND “angioedema” [tiab]. The latest search date was 15 December 2024. Only individual case reports and case series were considered, and conference abstracts were excluded. The following details were obtained for each case report: patient age, gender, amlodipine (dosage and duration of therapy), concomitant drugs and diseases, outcome, management of angioedema episodes, and interpretation of the causality assessment using the Naranjo algorithm where the scores were categorized into one of the following: definite (>9), probable (5–8), possible (1–4), and doubtful (<0) [25].

### 2.8. Statistical Analysis

We used descriptive statistics for summarizing the demographic variables, wherein the numerical variables were presented as means (SD) and the categorical variables as proportions (%) from the AERS ICSRs and published case reports. All statistical analyses were performed in SPSS© (IBM SPSS Statistics for Windows, Version 27.0; IBM Corp., Armonk, NY, USA).

## 3. Results

### 3.1. Search Results

**Figure 1:**
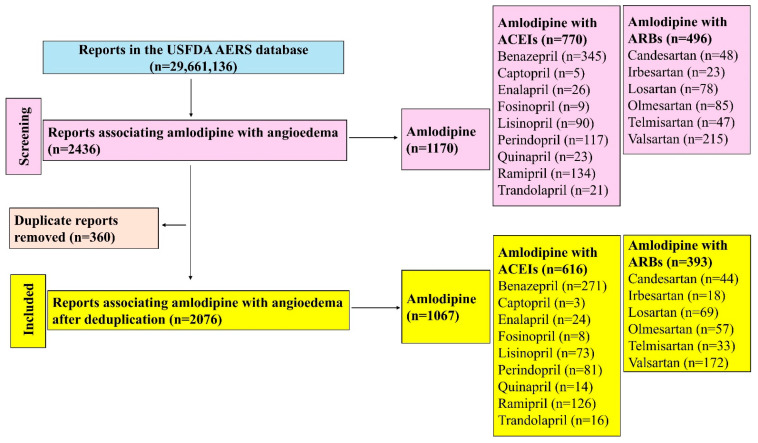
Study flow diagram. A total of 2076 reports relating to amlodipine associated with angioedema was included in this study.

A total of 29,661,136 reports were available in the database, of which 2076 reports (1067 with amlodipine and 1009 with amlodipine–RAAS-i drug combinations) were retrieved for assessing the risk of angioedema (Figure 1). Among the ACEIs in combination with amlodipine, the most reports exist for benazepril (n = 271), followed by ramipril (n = 126), while among the ARB combinations, valsartan (n = 172) followed by losartan (n = 69) had the most reports with angioedema.

A summary of the demographic characteristics of patients included in the reports is outlined in Table 2. Most patients were above 40 years of age with female predilection.

### 3.2. Signal Detection Measures

**Figure 2:**
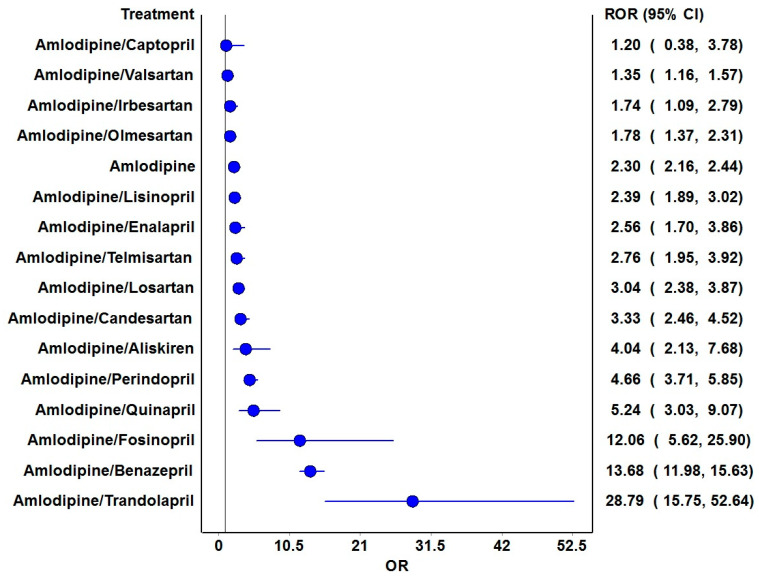
ROR plots for the risk of angioedema. The blue circles represent the point estimates of ROR, and the horizontal blue lines represent 95% confidence intervals for ROR. The black vertical line is the line of no significant difference in the risk of angioedema.

Table 3 lists the signal detection measures for the risk of angioedema with amlodipine. Both frequentist and Bayesian measures generated signals for amlodipine and its combination with the following drugs: aliskiren, ACEIs (benazepril, fosinopril, perindopril, quinapril, and trandolapril), and ARBs (candesartan and losartan). The ROR plots indicate an increased reporting ratio of angioedema with amlodipine and all combinations with ACEIs (except captopril) and ARBs and aliskiren (Figure 2).

### 3.3. Interaction Analysis

The results of the interaction analyses through multiplicative models are summarized in Table 4. No significant interaction signals were observed except for the combination of amlodipine with trandolapril.

### 3.4. Reported Outcomes for the Risk of Angioedema with Amlodipine

**Figure 3:**
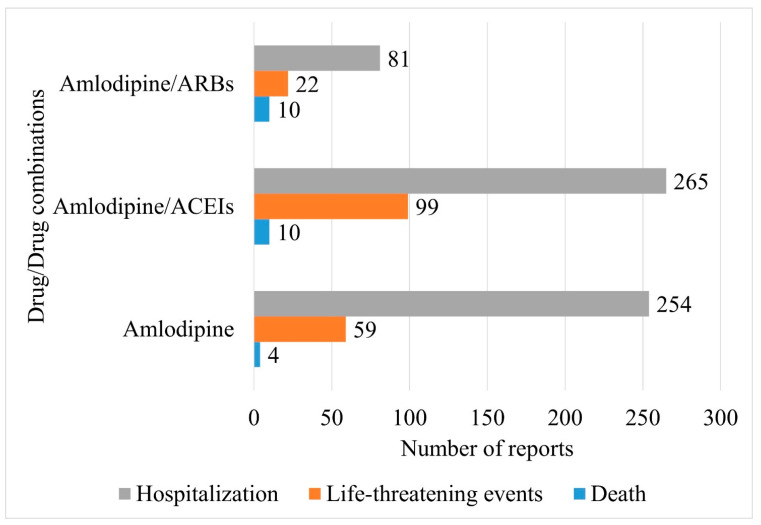
Comparison of outcomes reported for amlodipine-associated angioedema. The horizontal bar chart represents the distribution of outcomes between amlodipine and its combination with RAAS-i drugs for the risk of angioedema.

Distributions of key outcomes reported with amlodipine and its combinations with RAAS-i drugs are depicted in Figure 3. A significant difference was observed in the distribution of outcomes between the interventions (χ^2^: 23.7; df: 4; *p*-value: <0.0001).

### 3.5. Case Reviews

The search strategy led to twenty-four articles, of which seven [23,24,25,26,27,28,29] were related to amlodipine-associated angioedema (Table 5). The age ranged between 2.5 and 67 years, and the male/female ratio was 4:3. Five presented with oropharyngeal/facial edema, and one each had edema in the upper respiratory tract and gastrointestinal tract. Causality assessment revealed definite association of angioedema with amlodipine in two and possible association in the remaining cases. They were reported to be treated most with corticosteroids and antihistamines along with discontinuation of amlodipine. All patients recovered successfully from the angioedema episodes.

## 4. Discussion

### 4.1. Statement of Key Findings

The present study encompassing a comprehensive analysis of the USFDA AERS database, encompassing over 29 million reports, revealed several significant findings regarding amlodipine-associated angioedema. We identified 2076 relevant reports, with approximately half involving amlodipine monotherapy and half involving amlodipine–RAAS-i combinations. Both frequentist and Bayesian approaches generated positive safety signals for amlodipine alone and in combination with specific RAAS-i drugs, including aliskiren, several ACEIs, and certain ARBs. The interaction analysis revealed a significant signal only for the amlodipine–trandolapril combination. A review of published case reports demonstrated a diverse age distribution (2.5–67 years), with oropharyngeal/facial edema being the most common presentation. Causality assessment of these cases indicated definite association in two cases and possible association in others, with all patients achieving successful recovery following treatment with corticosteroids, antihistamines, and drug discontinuation. These findings suggest a potential safety concern regarding amlodipine-associated angioedema, particularly in specific drug combinations, warranting careful clinical consideration.

### 4.2. Comparison with Existing Literature

Our analysis demonstrates that amlodipine, both alone and in combination with RAAS-i drugs, is associated with an increased risk of angioedema. However, it is crucial to acknowledge that the diagnosis of drug-associated angioedema remains predominantly clinical, based on non-validated criteria, and lacks definitive objective biomarkers [33]. The pathophysiological mechanism underlying amlodipine-associated angioedema remains incompletely understood. While drug-associated angioedema typically manifests through either bradykinin-mediated or mast cell-mediated pathways [34], amlodipine’s mechanism appears to involve endothelial nitric oxide synthase (eNOS) activation, leading to nitric oxide production that can be antagonized by icatibant, a bradykinin-receptor antagonist [35]. This pathway’s potential significance is underscored by observations of elevated serum eNOS in patients with hereditary angioneurotic edema [36]. Amlodipine stimulation of eNOS was not observed with other calcium channel blockers and was observed to be related to a novel kinin-dependent pathway, which was also observed with an inactive enantiomer of amlodipine [37]. Further, amlodipine was observed to promote unclamping of eNOS from caveolin in endothelial cells, which potentiates the production of nitric oxide in response to agonist such as bradykinin [38]. Also, an in vitro study among cultured endothelial cells revealed potentiation of vascular endothelial growth factor-associated nitric oxide release by amlodipine by preventing the binding of acylated eNOS complex with caveolin [39]. Nitric oxide has been implicated in the pathogenesis of angioedema due to the resulting vasodilation [4,40]. Although bradykinin-receptor antagonists and kallikrein inhibitors are now approved therapeutic options for angioneurotic edema [41], suggesting a bradykinin-mediated mechanism, it is noteworthy that published case reports of amlodipine-associated angioedema document resolution primarily with corticosteroids and antihistamines rather than these newer agents. Recent research has revealed that 41% of patients with presumed ACEI/ARB-associated angioedema experienced recurrent episodes even after discontinuation, ultimately being diagnosed with mast cell-mediated angioedema, suggesting potential over attribution to RAAS-i drugs [42]. Our case report analysis identified two patients with recurrent angioedema following amlodipine rechallenge, establishing a definitive causal relationship. These findings emphasize the critical need for mechanistic studies to elucidate the precise pathophysiological pathway of amlodipine-associated angioedema.

Our interaction analysis revealed no significant potentiation of angioedema risk when combining amlodipine with most RAAS-i drugs, with the notable exception of the trandolapril combination. While we rigorously followed an established pharmacovigilance methodology and implemented thorough deduplication procedures, the possibility of residual duplicate reporting cannot be eliminated, potentially contributing to this isolated finding. The true clinical significance of this interaction warrants validation through comprehensive real-world studies comparing angioedema incidence across various monotherapy and combination therapy regimens involving RAAS-i drugs and amlodipine. Based on our current findings, we maintain that the established clinical benefits of combination therapy with amlodipine and RAAS-i drugs outweigh the potential risks, and clinicians should continue prescribing these combinations where clinically indicated, while maintaining appropriate vigilance for angioedema symptoms.

Given the potentially life-threatening nature of angioedema reactions, healthcare providers must maintain heightened awareness when initiating amlodipine therapy. For patients with a documented history of drug-induced angioedema, physicians should evaluate alternative antihypertensive options. Caution is warranted when prescribing amlodipine alongside medications with established angioedema risks, especially ACEIs and ARBs. In the absence of predictive laboratory markers for angioedema risk, comprehensive patient education and close monitoring are essential strategies for early recognition of amlodipine-associated angioedema episodes.

### 4.3. Strengths, Limitations, and Way Forward

This study presents several notable strengths, including its comprehensive analysis of a large pharmacovigilance database spanning two decades, employment of multiple signal detection algorithms, and integration of both spontaneous reports and published case reviews. However, certain limitations inherent to pharmacovigilance studies must be acknowledged. The USFDA AERS database is subject to underreporting, reporting bias, and lacks denominator data representing the total number of patients exposed to these medications. Additionally, the database may contain duplicate reports despite our careful deduplication efforts, and the quality of information in spontaneous reports can be variable. The voluntary nature of reporting means that not all adverse events are captured, and causality cannot be definitively established. Furthermore, important clinical information such as drug dosages, duration of therapy, and patient comorbidities are often incomplete or missing. Also, disproportionality analysis is useful only for hypothesis generation and not for hypothesis testing. Details about patients receiving drug/s of interest and not encountering angioedema were not available in the USFDA AERS database, precluding detailed subgroup analyses on either age/gender or incidence rate of angioedema. Hence, the findings must be interpreted cautiously and need to be validated in prospective studies. Moving forward, these findings should be validated through well-designed observational studies, particularly focusing on the identified drug combinations with significant signals. Future research should aim to elucidate the underlying mechanisms of amlodipine-associated angioedema and investigate potential risk factors that may predispose certain patients to this adverse event. Prospective studies examining the temporal relationship between drug initiation and angioedema onset, as well as the impact of dose modifications, would provide valuable insights for clinical practice. Furthermore, the relationship and the impact of concurrent drugs related to amlodipine-associated angioedema should be explored in future studies.

## 5. Conclusions

In conclusion, the present disproportionality analysis of the USFDA AERS database has identified potential safety signals for angioedema associated with amlodipine, both as monotherapy and in combination with specific RAAS-i drugs. While absolute risk appears modest, the widespread use of these medications necessitates clinical awareness of this potential adverse effect. The detection of safety signals, particularly with certain drug combinations, underscores the importance of vigilant monitoring, especially during the initial weeks of therapy. However, given the established cardiovascular benefits of these medications, our findings should not discourage their appropriate clinical use but rather promote informed prescribing decisions and enhanced patient monitoring. Future research should focus on elucidating the precise pathophysiological mechanisms, identifying susceptible patient populations, and developing targeted preventive strategies. Additionally, prospective studies comparing the real-world incidence of angioedema across various antihypertensive regimens would provide valuable insights for optimizing therapeutic approaches. Until more definitive evidence emerges, clinicians should maintain a balanced approach, weighing the well-documented benefits of these medications against the potential risk of angioedema, while ensuring prompt recognition and management of this adverse event.

## Figures and Tables

**Table 1:** Signal detection measure used for amlodipine–RAAS-i drugs for the risk of angioedema.

	Angioedema	All Adverse Events Except Angioedema	Total
Amlodipine with RAAS-i drug	n_111_	n_110_	n_11+_
Amlodipine without RAAS-i drug	n_101_	n_100_	n_10+_
RAAS-i drug without amlodipine	n_011_	n_010_	n_01+_
Neither amlodipine nor RAAS-i drug	n_001_	n_000_	n_00+_
Total	n_++1_	n_++0_	n_+++_
Signal detection for drug–drug interaction			
	Amlodipine	RAAS-i drugs	
Amlodipine	p_11_ (=n_111_/n_11+_)	p_10_ (=n_101_/n_10+_)	
RAAS-i drugs	p_01_ (=n_011_/n_01+_)	p_00_ (=n_001_/n_00+_)	
Log-linear regression for the risk of angioedema (e^β12^): (p_11_ × p_00_)/ (p_10_ × p_01_)			
Logistic regression for the risk of angioedema (e^γ12^): [p_11_/(1 − p_11_) × p_00_/(1 − p_00_)]/[p_10_/(1 − p_10_) × p_01_/(1 − p_01_)]			

n: number of reports; p: proportion of reports; and RAAS-I drugs: renin–angiotensin–aldosterone system interfering drugs.

**Table 2:** Demographic characteristics of patients in unique reports.

Characteristics		Amlodipine Without RAAS-i Drugs (n = 1067)	Amlodipine in Combination with RAAS-i Drugs	
			ACEIs (n = 616)	ARBs (n = 393)
Age Groups [n (%)]	<18	22 (2.1)	3 (0.5)	2 (0.8)
	>18 to <40	51 (4.8)	28 (4.5)	5 (2)
	>40 to <65	340 (31.9)	261 (42.4)	148 (37.7)
	>65	332 (31.1)	147 (23.8)	110 (28)
	Not specified	322 (30.2)	177 (28.7)	128 (32.6)
Quantitative age (years)	Mean (SD)	61.2 (17.5)	60.1 (13.4)	63 (13.5)
	Median (range)	63 (0–96)	59 (3–95)	62 (3–96)
Gender [n (%)]	Male	280 (26.2)	247 (40.1)	149 (37.9)
	Female	654 (61.3)	302 (49.3)	202 (51.4)
	Unknown	133 (12.5)	67 (10.6)	42 (10.7)
Reporting year [n (%)]	2004–2008	79 (7.4)	164 (26.6)	33 (8.4)
	2009–2012	196 (18.4)	53 (8.6)	75 (19.1)
	2013–2016	181 (17)	103 (16.7)	83 (21.1)
	2017–2020	308 (28.9)	124 (20.1)	68 (17.3)
	2021–2024 (September)	303 (28.4)	172 (27.9)	134 (34.1)
Reporting countries	USA	492 (46.1)	280 (45.5)	93 (23.7)
	Other countries and not reported	575 (53.9)	336 (54.5)	300 (72.3)

RAAS-i: renin–angiotensin–aldosterone system interfering; ACEIs: angiotensin-converting enzyme inhibitors; ARBs: angiotensin receptor blockers.

**Table 3:** Signal detection measures for the risk of angioedema.

Drug/s	RRR	PRR	Lower Limit of 95% CI of PRR	Upper Limit of 95% CI of PRR	χ^2^	Number of Reports	IC025	EBGM05
Amlodipine	2.2	2.2	2.1	2.4	747.6	1067	1.1	2.1 *
Combination with direct renin inhibitor								
Aliskiren	3.8	3.8	2.1	7	18.56	10	1	2.2 *
Combination with angiotensin-converting enzyme inhibitors								
Benazepril	11.2	11.2	10.1	12.4	2545.7	271	3.1	9.8 *
Captopril	1.2	1.2	0.4	3.7	0	3	0.1	0.4
Enalapril	2.5	2.5	1.7	3.7	20.4	24	0.9	1.7
Fosinopril	10.1	10.1	5.4	18.9	57.8	8	1.6	4.7 *
Lisinopril	2.3	2.3	1.9	2.9	55.1	73	1	1.8
Perindopril	4.4	4.4	3.6	5.4	211.4	81	1.7	3.5 *
Quinapril	5.9	5.9	3	8.1	40.1	14	1.3	2.8 *
Trandolapril	19.3	19.3	13	28.8	264.8	16	2.3	10.6 *
Combination with angiotensin receptor blockers								
Candesartan	3.2	3.2	2.4	4.3	65.5	44	1.2	2.4 *
Irbesartan	1.7	1.7	1.1	2.7	4.8	18	0.5	1.1
Losartan	2.9	2.9	2.3	3.7	87.5	69	1.2	2.3 *
Olmesartan	1.8	1.8	1.4	2.3	18	57	0.6	1.3
Telmisartan	2.7	2.7	1.9	3.7	33.6	33	1	1.9
Valsartan	1.3	1.3	1.2	1.6	14.7	172	0.4	1.2

RRR: relative reporting ratio; PRR: proportional reporting ratio; χ^2^: chi-square statistics value; IC: information component; EBGM: empirical Bayes geometric mean; and *: signals detected by both frequentist and Bayesian methods.

**Table 4:** Assessment of interaction analysis between amlodipine and RAAS-i drugs for the risk of angioedema.

RAAS-i Drug Combination with Amlodipine	e^β12^	e^γ12^
Combination with direct renin inhibitor		
Aliskiren	0.4	0.4
Combination with angiotensin-converting enzyme inhibitors		
Benazepril	0.4	0.3
Captopril	0.1	0.1
Enalapril	0.1	0.1
Fosinopril	0.2	0.2
Lisinopril	0.1	0.03
Perindopril	0.2	0.2
Quinapril	0.4	0.4
Ramipril	0.2	0.2
Trandolapril	1.8 *	2.5 *
Combination with angiotensin receptor blockers		
Candesartan	0.7	0.7
Irbesartan	0.3	0.3
Losartan	0.3	0.3
Olmesartan	0.7	0.7
Telmisartan	0.5	0.5
Valsartan	0.5	0.5

e^β12^: log (risk of event) by log-linear regression; e^γ12^: logit (risk of event) by logistic regression; and *: statistically significant.

**Table 5:** Characteristics of cases included in the case reports.

Report ID	Age (Years)	Gender	Amlodipine: Dose and Duration	Clinical Presentation	Concomitant Medications	Concomitant Diseases	Outcome of Angioedema Episode	Treatment of Angioedema	Causality Assessment
Hom 2012 [26]	2.5	Male	Not mentioned; 1.25 years	Stridor, swelling of arytenoids and glottis	Immunosuppressive drugs (exact details were not specified)	Stage 4 hepatoblastoma	Recovery	Amlodipine discontinuation; dexamethasone and epinephrine were administered	Possible
Kuruvila 2018 [27]	67	Female	5 mg once daily; 2 weeks	Periorbital and lip edema	Hydralazine, metoprolol, atorvastatin, and furosemide	Congestive heart failure; past history of ACEI-associated angioedema		Amlodipine discontinuation; corticosteroids and antihistamines were administered	Definite (patient developed angioedema following rechallenge with amlodipine)
Morgenthau 2019 [28]	50	Male	Not mentioned; 4–6 months	Swelling of jaws, tongue and lips	Lisinopril, allopurinol, venlafaxine, and tolvaptan	Polycystic kidney disease		Amlodipine and lisinopril discontinuation; corticosteroids, epinephrine, ranitidine, and diphenhydramine were administered	Possible
Pierce 2011 [29]	8	Male	5 mg once daily; 18 days	Tongue swelling	Nicardipine intravenously for 3 days prior to oral amlodipine; chemotherapeutic drugs for lymphoma	Burkitt lymphoma		Amlodipine discontinued	Possible
Russo 2023 [30]	38	Male	Not mentioned; 6–8 weeks	Lip swelling	Multivitamin	None		Amlodipine discontinued; dexamethasone, diphenhydramine, famotidine, tranexamic acid were administered. Considering no improvement, the patient received additional dose of famotidine and started on methylprednisolone.	Possible
Southward 2009 [31]	50	Female	10 mg; 1 day	Face and tongue swelling	Clonidine, valsartan, verapamil, metoprolol, ranitidine, cinacalcet, nicardipine, fosphenytoin, fentanyl, propofol, famotidine and vancomycin	Bronchial asthma, chronic kidney disease, left hemiplegia		Amlodipine discontinued; diphenhydramine, hydrocortisone, and ranitidine were administered	Possible
Turcu 2009 [32]	56	Female	Not mentioned	Abdominal pain. Radiological examination revealed multiple prominent mural thickening of different intestinal segments, from the duodenum to the colon, but always involving the terminal ileum.	Atorvastatin, furosemide, metoprolol, irbesartan, clonidine, aspirin, fenofibrate, pioglitazone, glimepiride, insulin, iron, bupropion, epoetin alpha, and multivitamins	Type II diabetes, hypercholesterolemia, lactose intolerance, breast cancer, and depression		Amlodipine and irbesartan were discontinued	Definite (rechallenge was positive)

ACEI: angiotensin–converting enzyme inhibitor.

## Data Availability

The data are available in the USFDA AERS web-portal that can be accessed as follows: https://fis.fda.gov/sense/app/95239e26-e0be-42d9-a960-9a5f7f1c25ee/sheet/7a47a261-d58b-4203-a8aa-6d3021737452/state/analysis (accessed on 14 December 2024).

## Supplementary Materials

The following Supporting Information can be downloaded at: https://www.mdpi.com/article/10.3390/jcm14041097/s1.
