# Supplementary material for: Amlodipine-Associated Angioedema: An Integrated Pharmacovigilance Assessment Using Disproportionality and Interaction Analysis and Case Reviews
Source: J Clin Med. 2025 Feb 8;14(4):1097. doi: 10.3390/jcm14041097 (PMC11856106; doi:10.3390/jcm14041097)
Supplement: Supplementary file 1 [file jcm-14-01097-s001.zip › jcm-3444053-supplementary.pdf]

Electronic Supplementary Table S1. The list of Preferred Terms included in the SMQ (Narrow) for angioedema.

Preferred Term	MedDRA code
Acquired C1 inhibitor deficiency	10081035
Allergic oedema	10060934
Angioedema	10002424
Circumoral oedema	10052250
Circumoral swelling	10081703
Conjunctival oedema	10010726
Corneal oedema	10011033
Epiglottic oedema	10015029
Eye oedema	10052139
Eye swelling	10015967
Eyelid oedema	10015993
Face oedema	10016029
Gingival oedema	10049305
Gingival swelling	10018291
Gleich's syndrome	10066837
Hereditary angioedema	10019860
Hereditary angioedema with C1 esterase inhibitor deficiency	10080955
Hereditary angioedema with normal C1 esterase inhibitor	10080953
Idiopathic angioedema	10073257
Idiopathic histaminergic angioedema	10088780
Idiopathic urticaria	10021247
Intestinal angioedema	10076229
Laryngeal oedema	10023845
Laryngotracheal oedema	10023893
Limbal swelling	10070492
Lip oedema	10024558
Lip swelling	10024570
Mouth swelling	10075203
Oculorespiratory syndrome	10067317
Oedema mouth	10030110
Oropharyngeal oedema	10078783
Oropharyngeal swelling	10031118
Palatal oedema	10056998
Palatal swelling	10074403
Periorbital oedema	10034545
Periorbital swelling	10056647
Pharyngeal oedema	10034829
Pharyngeal swelling	10082270
Respiratory tract oedema	10070774

Scleral oedema	10057431
Swelling face	10042682
Swelling of eyelid	10042690
Swollen tongue	10042727
Tongue oedema	10043967
Tracheal oedema	10044296
Urticaria	10046735
Urticaria cholinergic	10046740
Urticaria chronic	10052568
Urticaria papular	10046750

Electronic Supplementary Table S2. Search strategy employed in this study.

Amlodipine	**Drugs:** Amlodipine AND NOT aliskiren AND NOT benazepril AND NOT captopril AND NOT enalapril AND NOT fosinopril AND NOT lisinopril AND NOT perindopril AND NOT quinapril AND NOT ramipril AND NOT trandolapril AND NOT candesartan AND NOT irbesartan AND NOT losartan AND NOT olmesartan AND NOT telmisartan AND NOT valsartan<br><br>**Role of the drug:** Primary suspect<br><br>**Adverse event:** SMQ (Narrow): Angioedema
Amlodipine with aliskiren	**Drugs:** Amlodipine AND aliskiren<br><br>**Role of the drug:** Primary suspect (for amlodipine)<br><br>**Adverse event:** SMQ (Narrow): Angioedema
Amlodipine with benazepril	**Drugs:** Amlodipine AND benazepril<br><br>**Role of the drug:** Primary suspect (for amlodipine)<br><br>**Adverse event:** SMQ (Narrow): Angioedema
Amlodipine with captopril	**Drugs:** Amlodipine AND captopril<br><br>**Role of the drug:** Primary suspect (for amlodipine)<br><br>**Adverse event:** SMQ (Narrow): Angioedema
Amlodipine with enalapril	**Drugs:** Amlodipine AND enalapril<br><br>**Role of the drug:** Primary suspect (for amlodipine)<br><br>**Adverse event:** SMQ (Narrow): Angioedema
Amlodipine with enalaprilat	**Drugs:** Amlodipine AND enalaprilat<br><br>**Role of the drug:** Primary suspect (for amlodipine)<br><br>**Adverse event:** SMQ (Narrow): Angioedema
Amlodipine with fosinopril	**Drugs:** Amlodipine AND fosinopril<br><br>**Role of the drug:** Primary suspect (for amlodipine)<br><br>**Adverse event:** SMQ (Narrow): Angioedema
Amlodipine with lisinopril	**Drugs:** Amlodipine AND lisinopril<br><br>**Role of the drug:** Primary suspect (for amlodipine)

	**Adverse event:** SMQ (Narrow): Angioedema
Amlodipine with moexipril	**Drugs:** Amlodipine AND moexipril<br><br>**Role of the drug:** Primary suspect (for amlodipine)<br><br>**Adverse event:** SMQ (Narrow): Angioedema
Amlodipine with perindopril	**Drugs:** Amlodipine AND perindopril<br><br>**Role of the drug:** Primary suspect (for amlodipine)<br><br>**Adverse event:** SMQ (Narrow): Angioedema
Amlodipine with quinapril	**Drugs:** Amlodipine AND quinapril<br><br>**Role of the drug:** Primary suspect (for amlodipine)<br><br>**Adverse event:** SMQ (Narrow): Angioedema
Amlodipine with ramipril	**Drugs:** Amlodipine AND ramipril<br><br>**Role of the drug:** Primary suspect (for amlodipine)<br><br>**Adverse event:** SMQ (Narrow): Angioedema
Amlodipine with trandolapril	**Drugs:** Amlodipine AND trandolapril<br><br>**Role of the drug:** Primary suspect (for amlodipine)<br><br>**Adverse event:** SMQ (Narrow): Angioedema
Amlodipine with azilsartan	**Drugs:** Amlodipine AND azilsartan<br><br>**Role of the drug:** Primary suspect (for amlodipine)<br><br>**Adverse event:** SMQ (Narrow): Angioedema
Amlodipine with candesartan	**Drugs:** Amlodipine AND candesartan<br><br>**Role of the drug:** Primary suspect (for amlodipine)<br><br>**Adverse event:** SMQ (Narrow): Angioedema
Amlodipine with eprosartan	**Drugs:** Amlodipine AND eprosartan<br><br>**Role of the drug:** Primary suspect (for amlodipine)<br><br>**Adverse event:** SMQ (Narrow): Angioedema
Amlodipine with irbesartan	**Drugs:** Amlodipine AND irbesartan<br><br>**Role of the drug:** Primary suspect (for amlodipine)

	**Adverse event:** SMQ (Narrow): Angioedema
Amlodipine with losartan	**Drugs:** Amlodipine AND losartan<br><br>**Role of the drug:** Primary suspect (for amlodipine)<br><br>**Adverse event:** SMQ (Narrow): Angioedema
Amlodipine with olmesartan	**Drugs:** Amlodipine AND olmesartan<br><br>**Role of the drug:** Primary suspect (for amlodipine)<br><br>**Adverse event:** SMQ (Narrow): Angioedema
Amlodipine with telmisartan	**Drugs:** Amlodipine AND telmisartan<br><br>**Role of the drug:** Primary suspect (for amlodipine)<br><br>**Adverse event:** SMQ (Narrow): Angioedema
Amlodipine with valsartan	**Drugs:** Amlodipine AND valsartan<br><br>**Role of the drug:** Primary suspect (for amlodipine)<br><br>**Adverse event:** SMQ (Narrow): Angioedema
